# In Vivo Optical Imaging of Interscapular Brown Adipose Tissue with ^18^F-FDG via Cerenkov Luminescence Imaging

**DOI:** 10.1371/journal.pone.0062007

**Published:** 2013-04-24

**Authors:** Xueli Zhang, Chaincy Kuo, Anna Moore, Chongzhao Ran

**Affiliations:** 1 Molecular Imaging Laboratory, MGH/MIT/HMS Athinoula A. Martinos Center for Biomedical Imaging, Department of Radiology, Massachusetts General Hospital/Harvard Medical School, Charlestown, Massachusetts, United States of America; 2 Center for Drug Discovery, School of Pharmacy, China Pharmaceutical University, Nanjing, China; 3 Caliper, a Perkin Elmer Company, Alameda, California, United States of America; Wayne State University, United States of America

## Abstract

**Objective:**

Brown adipose tissue (BAT), a specialized tissue for thermogenesis, plays important roles for metabolism and energy expenditure. Recent studies validated BAT’s presence in human adults, making it an important re-emerging target for various pathologies. During this validation, PET images with ^18^F-FDG showed significant uptake of ^18^F-FDG by BAT under certain conditions. Here, we demonstrated that Cerenkov luminescence imaging (CLI) using ^18^F-FDG could be utilized for *in vivo* optical imaging of BAT in mice.

**Methods:**

Mice were injected with ^18^F-FDG and imaged 60 minutes later with open filter and 2 minute acquisition. *In vivo* activation of BAT was performed by norepinephrine and cold treatment under isoflurane or ketamine anesthesia. Spectral unmixing and 3D imaging reconstruction were conducted with multiple-filter CLI images.

**Results:**

1) It was feasible to use CLI with ^18^F-FDG to image interscapular BAT in mice, with the majority of the signal (>85%) at the interscapular site originating from BAT; 2) The method was reliable because excellent correlations between *in vivo* CLI, *ex vivo* CLI, and *ex vivo* radioactivity were observed; 3) CLI could be used for monitoring BAT activation under different conditions; 4) CLI signals from the group under short-term isoflurane anesthesia were significantly higher than that from the group under long-term anesthesia; 5) The CLI spectrum of ^18^F-FDG with a peak at 640 nm in BAT after spectral unmixing reflected the actual context of BAT; 6) Finally 3D reconstruction images showed excellent correlation between the source of the light signal and the location and physical shape of BAT.

**Conclusion:**

CLI with ^18^F-FDG is a feasible and reliable method for imaging BAT in mice. Compared to PET imaging, CLI is significantly cheaper, faster for 2D planar imaging and easier to use. We believe that this method could be used as an important tool for researchers investigating BAT.

## Introduction

Brown adipose tissue (BAT) is a specialized tissue for thermogenesis in mammals, and it has been considered as a furnace in the body for burning excess calories. The function of BAT in mammals is to dissipate large amounts of chemical/food energy as heat, thus maintaining the energy balance of the whole body [1], [2], [3]. Investigations of BAT have been ongoing for decades, particularly using animals. It has been considered that BAT disappears from the body of adults and has no significant physiological relevance in adult humans [1], [4], [5], [6]. However, recent rediscovery of BAT in human adults by PET-CT scan has produced a new driving force for BAT studies [7], [8], [9], [10], [11], [12], [13], [14]. Multiple studies have demonstrated that BAT mass levels inversely correlate with body-mass index (BMI), and that physical exercises could increase BAT mass, suggesting that BAT may play important roles in obesity and diabetes [7], [8], [15], [16], [17]. In addition, BAT has emerged as an important target for various other diseases such as neurodegenerative disease and cancer [5], [9], [10], [11], [12]. Clearly, the ability to image BAT non-invasively would aid in clinical staging of various diseases as well as in drug development processes.

**Figure 1:**
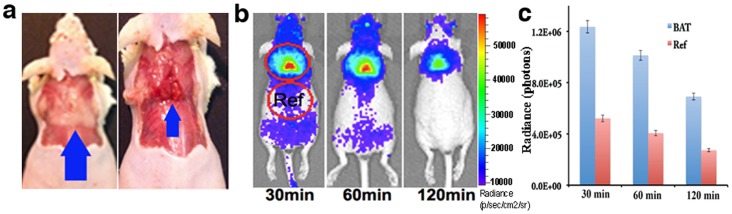
CLI imaging of the interscapular BAT. (a) Triangular contour of interscapular BAT (blue arrow) in a mouse; (left) BAT is covered with white adipose tissue, and (right) BAT is exposed. (b) CLI images of a mouse at 30, 60, 120 minutes after ^18^F-FDG (10.3 MBq) intravenous injection. The images clearly outline the contour of BAT, even after 120 minutes of ^18^F-FDG injection. (c) Quantitative analysis of CLI signal from interscapular BAT area and a reference area.

**Figure 2:**
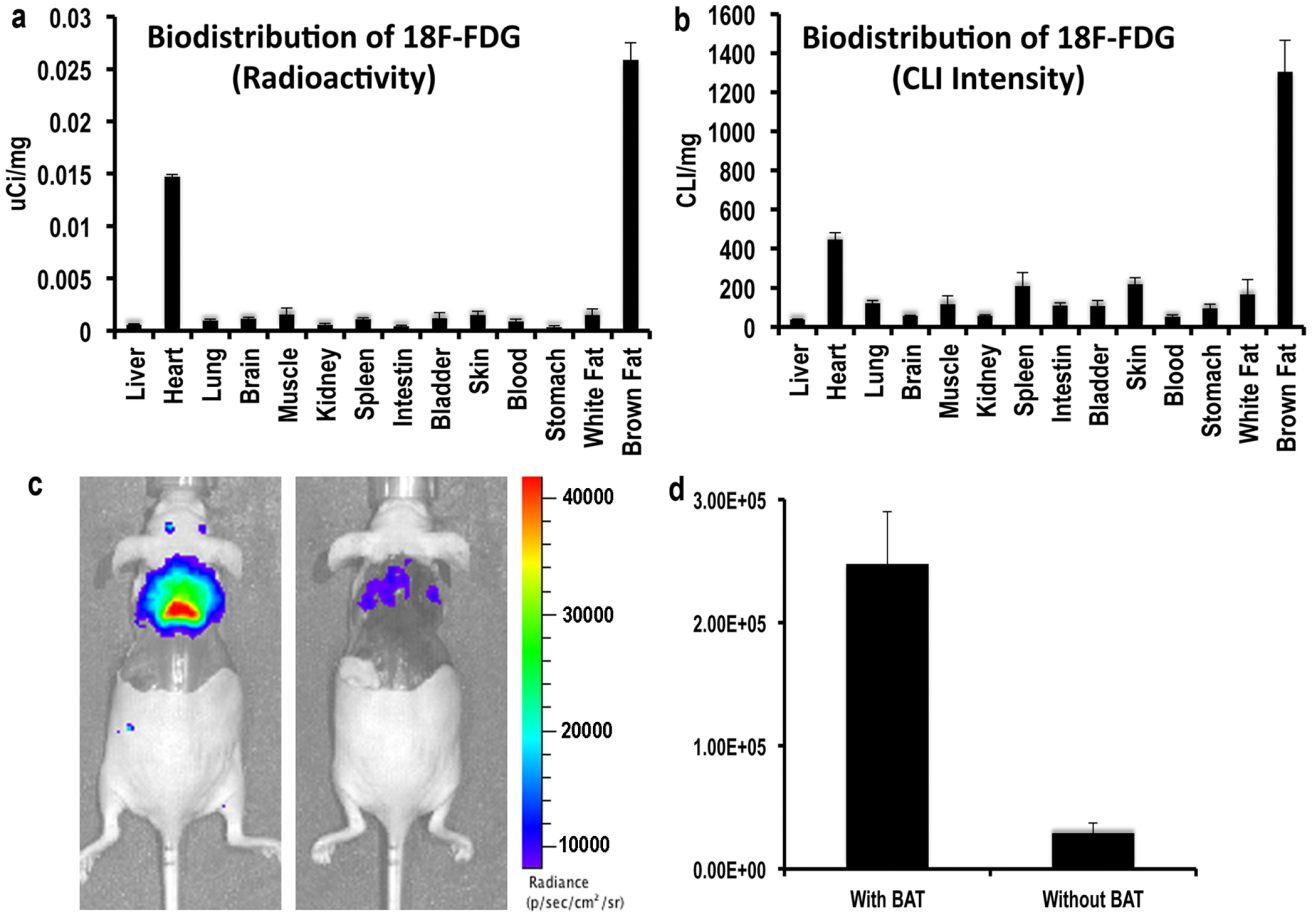
Validation of CLI signal originating from BAT (n = 3). (a–b) Biodistribution of ^18^F-FDG in dissected tissues. Radioactivity quantifications (a) and CLI readings (b). Both methods indicated that BAT had the highest ^18^F-FDG uptake. (c–d) Representative images of mice before (c, left) and after (c, right) BAT removal; (d) Quantification indicated that >85% CLI originated from BAT.

Cerenkov luminescence imaging (CLI) is a newly emerged molecular imaging technology [18], [19], [20], [21], [22], [23], [24], [25], [26], [27]. It utilizes luminescence generated from the β^+^ and β^−^ decay of radionuclides such as ^18^F and ^131^I in the medium. As a charged particle (such as β^+^ and β^−^) travels, it polarizes molecules in the medium [18], [19], [20]. When the polarized molecules relax back to equilibrium they emit electromagnetic waves. As the particle moves along its path, the waves destructively interfere with each other and no radiation is observed. However, if the particle velocity *v* is greater than the speed of light in the medium (*c/n*, where *c* is the speed of light, and *n* is the index of refraction), then a shockfront comprised from constructive interference is observed as Cerenkov radiation. The intensity of the emitted Cerenkov photons is proportional to the kinetic energy of the charged particle, in this case, the β^+/−^ ejected from the radionuclide [28]. As the condition that *v>c/n* in the medium, the energy threshold necessary for Cerenkov light production varies for different media, dependent on the index of refraction. [19], [28]. In addition, the spectrum of the radiation-luminescence consists of continuous wavelengths throughout the ultraviolet (UV) and visible spectrum [18], [19], [20], with the intensity distribution inversely proportional to the square of the wavelength. It is conceivable that the light emitted from the radionuclide with a shorter wavelength could be used a replacement of ultraviolet (UV) light, while the longer wavelength light could be used for *in vivo* imaging due to its superior tissue penetration. We have previously demonstrated that the UV portion of Cerenkov luminescence could be utilized for *in vivo* photoactivation of caged luciferin [21], while Dothager and Liu utilized this approach for quantum dots excitation [23], [29]. Others have reported that CLI could be a reliable tool for imaging tumors, assisting intraoperative surgical resection, endoscopic imaging, and for monitoring therapeutic effectiveness of anticancer drugs [18], [30], [31], [32], [33], [34]. Several excellent reviews have summarized the advantages and disadvantages of this technology [26], [35], [36]. Recently, Spinelli and Ackerman also demonstrated that Cerenkov luminescence from alpha-emitter daughter products could be used for imaging [37], [38]. In this report, we demonstrated that CLI could be used for BAT imaging. BAT is an ideal target for CLI due to the following reasons: 1) Location of BAT in mice is unique since it is situated away from large organs such as liver, heart, and stomach, and thus signal interference from these large organs is minimal (Fig. 1a); 2) BAT location is shallow, which allows for more photons to reach the detection camera; 3) BAT is a whole mass organ; 4) BAT has a unique triangular physical shape which is easy to distinguish from other tissues (Fig. 1a).

PET (positron emission tomography) imaging confirmed the significant uptake of ^18^F-FDG in BAT under certain conditions in humans [7], [8], [10], [13], [39]. Previous animal studies also demonstrated that ^18^F-FDG could be significantly taken up by BAT in mice [40], [41]. In this report, we hypothesized that BAT could be optically imaged with ^18^F-FDG utilizing Cerenkov luminescence imaging.

For small animal research, we believe that CLI imaging of BAT could be a useful complementary method for PET imaging with ^18^F-FDG, particularly for laboratories without an accessible PET scanner. Additionally, CLI imaging with an optical imaging system is cheaper and therefore, more cost effective. For simple 2D planar imaging it could be faster than PET imaging, and could potentially be used for high throughput screening. Apart from the ability to image BAT, our study also serves as an additional validation of CLI as an emerging imaging modality.

## Results

### 1. CLI Feasibility for BAT Imaging

To test whether CLI with ^18^F-FDG is capable of providing significant signal and contrast, 10.3 MBq of ^18^F-FDG was intravenously injected into nude mice. For each time point, mice were anesthetized by isoflurane, and the CLI images were acquired within 3 minutes after induction of anesthesia using a whole-body optical imaging system. No stimulation for BAT activation was applied. We found that CLI signals from interscapular BAT (Fig. 1a) were remarkably bright at all time points (30, 60, 120 minutes), and the contrast between BAT and the adjacent reference area was apparent (Fig. 1b). Signal ratios of BAT and the reference area were 2.37-, 2.49-, and 2.53-fold respectively at 30, 60, and 120 minutes after injection. Interestingly, contours of BAT images closely resembled its physical appearance (interscapular BAT of rodents consists of two lobes that make a triangle contour) (Fig. 1a). These results suggested that CLI imaging of BAT with ^18^F-FDG was feasible.

### 2. Validation of the CLI Signal from the Interscapular Area

To confirm whether the majority of the interscapular CLI signal was from BAT, we first conducted biodistribution studies with CLI signal and radioactivity countings from the dissected tissues. Both CLI and radioactivity countings indicated that the heart and BAT were the two primary tissues for ^18^F-FDG uptake, with BAT displaying the highest uptake (Fig. 2a,b, and Fig. S1). Because the heart is located more deeply than the interscapular BAT when images were taken from the dorsal side, we speculated that the majority of the CLI signal in the interscapular area originated from BAT. To further validate this speculation, we imaged mice before and after BAT removal, and compared the images. We found that the majority of the signal (>85%) at the interscapular site originated from BAT (Fig. 2c,d). The residual signal was located near the upper rim of BAT and was most likely from the remnants of BAT tissue and other surrounding tissues, such as blood vessels, muscles, and bones.

### 3. CLI Reliability Study

**Figure 3:**
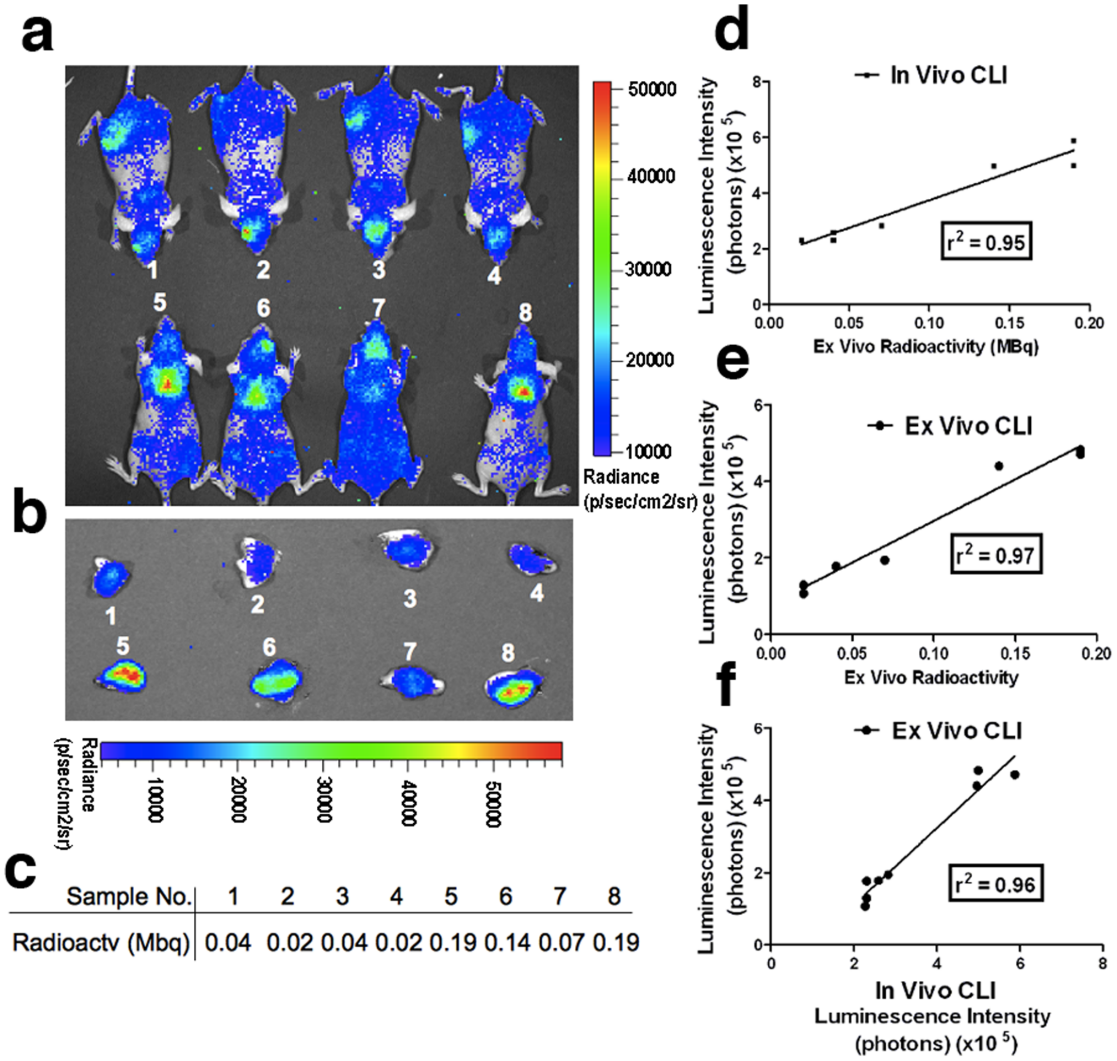
Reliability correlation studies of *in vivo* CLI imaging, *ex vivo* CLI imaging, and radioactive dosimetric countings of BAT. (a) *In vivo* CLI images of mice injected with ^18^F-FDG under ketamine/xylazine anesthesia without (top panel, mice #1–4) and with NE treatment (lower panel, mice #5–8). (b) *Ex vivo* CLI images of BAT of mice in (a). (c) Dosimetric countings of *ex vivo* BAT in (b). (d) Correlation between *in vivo* CLI signals and dosimetric countings. (e) Correlation between *ex vivo* CLI signals and dosimetric countings. (f) Correlation between *ex vivo* CLI signals and *in vivo* CLI signals. Correlation plots in D-F suggest that *in vivo* CLI signals, *ex vivo* CLI signals, and *ex vivo* radioactivity measurements were highly interrelated.

Since CLI imaging is still in the developmental stage, cross-validation of *in vivo* CLI signals with *ex vivo* CLI signals, as well as with its actual radioactivity, was necessary. It is known that activation of BAT by cold exposure or drugs (such as norepinephrine) is often needed to obtain reliable PET images [8], [10], [39], [40]. In our studies, we used similar methods to show the reliability of Cerenkov luminescence imaging. We used norepinephrine (NE) as a stimulator in these studies. Mice were stimulated with NE followed by *in vivo* CLI with ^18^F-FDG. Immediately after imaging, mice were sacrificed and BATs were collected and subjected to *ex vivo* CLI imaging and radioactivity measurements with a dosimeter. As expected, *in vivo* images of the NE- treated group showed significantly brighter signals than that of the non-treated control group (p<0.005, Fig. 3a and Fig. S2a). *Ex vivo* CLI quantitation and dosimetry countings displayed significantly higher values for the NE-treated group than for controls as well (p<0.01, Fig. 3b–c, and Fig. S2b–c). To further validate whether *in vivo* CLI signals reliably reflected the amount of radioactivity caused by ^18^F-FDG accumulation in BAT, linear regression fitting was used to evaluate a correlation between *in vivo* CLI, *ex vivo* CLI, and *ex vivo* dosimetry countings. We found that there was an excellent correlation between signals from *in vivo* CLI and *ex vivo* radioactivity (r^2^ = 0.95) (Fig. 3d), as well as between signals from *ex vivo* CLI and *ex vivo* radioactivity (r^2^ = 0.97) (Fig. 3e). Due to the depth-penetrating limitation of optical imaging, disparity between *in vivo* and *ex vivo* data often exists. However, our data demonstrated a good correlation between *in vivo* CLI and *ex vivo* CLI (r^2^ = 0.96) (Fig. 3f). Taken together, our data suggest that *in vivo* CLI imaging of BAT is reliable.

### 4. Monitoring BAT Activity using CLI

**Figure 4:**
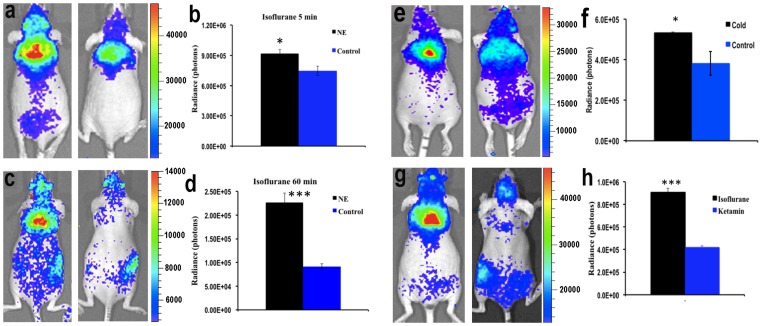
CLI imaging with ^18^F-FDG for monitoring BAT activation. (a) Representative CLI images of BAT of mice with (left) and without (right) NE stimulation under short isoflurane anesthesia. (b) Quantitative analysis of the CLI signals from the two groups in a (n = 4 for each group). (c) Representative CLI images of BAT of mice with (left) and without (right) NE treatment under long isoflurane anesthesia (60 minutes). (d) Quantitative analysis of the CLI signals from the two groups shown in c (n = 3–4 for each group). (e) Representative CLI images of BAT of mice with (left) and without (right) cold stimulation under short isoflurane anesthesia. (f) Quantitative analysis of the CLI signals from the two groups shown in e (n = 3–4 for each group). (g) Representative CLI images of BAT at 60 minutes after ^18^F-FDG injection under short isoflurane anesthesia (5 min) (left) and ketamine/xylazine anesthesia (70 minutes) (right). (h) Quantitative analysis of the CLI signals from the two groups shown in g (n = 4 for each group).

After confirmation of CLI feasibility and reliability for BAT imaging with ^18^F-FDG, we utilized this method for monitoring BAT activity. NE treatment and cold exposure are the most used methods for BAT activation [8], [10], [39], [40]. First, we compared BAT activity in the same group of mice with and without NE treatment under short (5 minute) isoflurane anesthesia. As expected, we found that BAT activity under NE-treated condition was significantly higher (1.23-fold) than without NE treatment (Fig. 4a, b). Similar results were observed under the long (60 minute) isoflurane anesthesia. Interestingly, the difference in BAT activity between NE-treated and non-treated animals was even larger under long isoflurane anesthesia (60 minute) than under short (5 min) isoflurane anesthesia (2.47 fold, Fig. 4c, d). PET imaging of human subjects injected with ^18^F-FDG revealed that the uptake of ^18^F-FDG in BAT was higher under cold exposure than at room temperature [3], [8], [42]. In this report, we found that there was a 39% increase of ^18^F-FDG uptake in the BAT of animals stimulated with cold exposure (Fig. 4e, f).

It has also been reported that different anesthesia regimens have significant effect on BAT activity [10], [41], [43]. Fueger et al. reported that ^18^F-FDG uptake in BAT was significantly reduced after 1 hour of ketamine anesthesia [41]. In the next study, we compared CLI signal of ^18^F-FDG accumulation in mice under different anesthesia regimens. We compared CLI signal from mice subjected to isoflurane or ketamine/xylazine anesthesia. One group of mice was subjected to isoflurane anesthesia for only for a short period time needed for injection and imaging (about 5 minutes). After complete clearance of the previous ^18^F-FDG dose, the same group was imaged again under ketamine/xylazine anesthesia for about 70 minutes. Our imaging data revealed that there was a 54% decrease in ^18^F-FDG BAT uptake in the group anesthetized with ketamine compared to the isoflurane group (Fig. 4g, h). Our results were similar to Fueger’s report, in which ^18^F-FDG uptake by BAT was dramatically depressed under long anesthesia treatment [41].

### 5. Spectral Unmixing and Multispectral Cerenkov Luminescence Tomography

**Figure 5:**
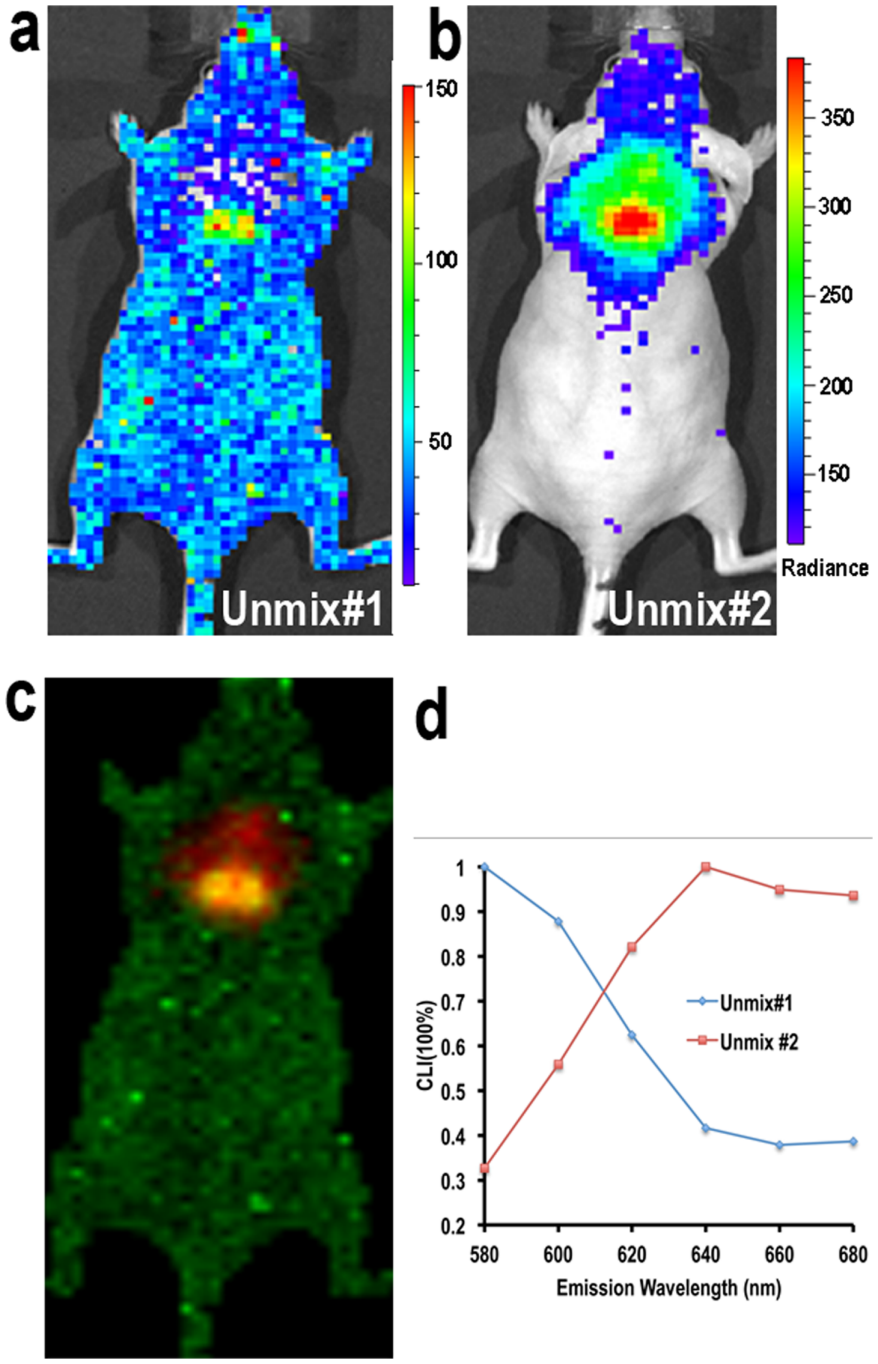
Spectral unmixing for unspecific CLI and BAT CLI. (a) Unmix #1 showed that unspecific CLI signal from ^18^F-FDG was distributed over the whole body (the unmixed spectrum for this image is shown in (d) (blue line)). (b) Unmix #2 indicated that the majority of CLI at interscapular site was from BAT (the unmixed spectrum is shown in (d) (red line)). CLI peak was around 640 nm. (c) Merged image of Unmix #1 and #2. (d) The CLI spectra of Unmix #1 and #2.

In this report, we collected CLI signal with multiple filters, and these data were used for spectral unmixing and multispectral Cerenkov luminescence tomography [31]. ^18^F-FDG (11.1 MBq) was injected in mice and multispectral images of the animals’ dorsal side were acquired 60 minutes after the injection with 20 nm-wide filters at 580, 600, 620, 640, 660 and 680 nm (raw images are shown in Fig. S3). Spectral unmixing was conducted with commercial software package to separate two components (BAT and unspecific signals). From Fig. 5a, it is clear that the unmixed component #1 (Unmix #1) represented unspecific CLI from ^18^F-FDG, with no particular area highlighted. The spectrum (blue line in Fig. 5d) further confirmed that the Unmix #1 represented an unspecific CLI signal, because the spectrum matched with the emission spectrum of ^18^F in pure media, in which the CLI intensity is inversely correlated with the square of the wavelength [18], [19], [20]. This indicates that the Cerenkov emissions in Unmix #1 are from very shallow depths, such as ^18^F-FDG accumulation in the skin, as the observed emission spectrum is not affected by tissue absorption. Unmixed component #2 (Unmix #2) reflected the signals from interscapular BAT (Fig. 5b). Notably, the spectrum peak (red line) was around 640 nm and the signal considerably inflated with the increase of the wavelength before reaching the peak, while the intensity of CLI was not dramatically decreased once it reached the peak (red line in Fig. 5d). This phenomenon is probably due to two facts: 1) the light absorption of hemoglobin in blood and cytochrome c in mitochondria for short wavelength CLI (<640 nm;BAT contains more mitochondria and is highly vascularized compared to other nearby tissues [1]); 2) better tissue penetration of the emitted light with longer wavelength.

**Figure 6:**
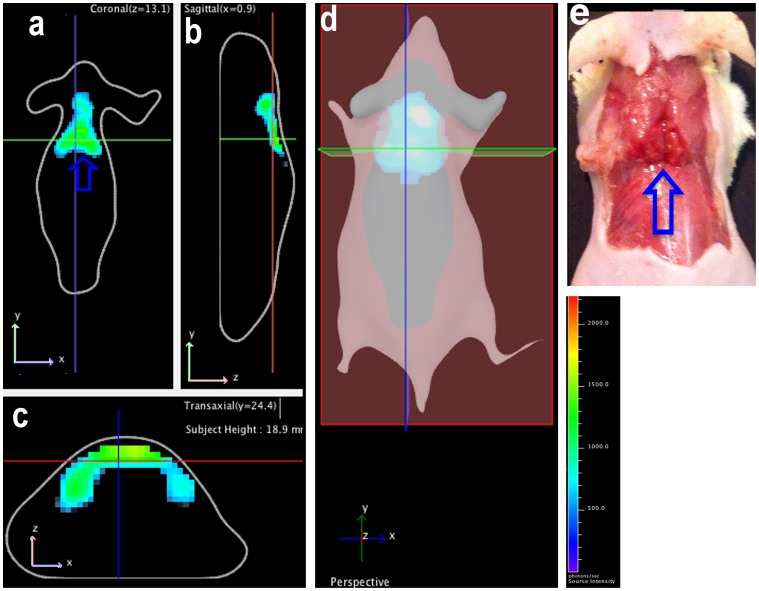
Multispectral Cerenkov luminescence tomography. (a–d) 3D reconstruction of the images. Interscapular BAT could be seen in coronal (a), sagittal (b), and transverse views (c), as well as in the 3D image (d); (e) Physical BAT shape is shown (blue arrow) which correlated with reconstructed images.

The 3D reconstruction was conducted with multispectral Cerenkov luminescence tomography (msCLT), which is based on a set of 2D planar CLI images acquired using a number of narrow bandpass filters. 3D image reconstruction utilizes the distinctive diffuse information at each wavelength [31], [44]. The advantage of this method is that it provides depth resolution and localization without requiring multiple views of the animal. Recently, several other groups have also demonstrated the feasibility of 3D CLI reconstruction [30], [45], [46], [47]. In the process of msCLT, the 1*/λ* ^2^ wavelength dependence of the Cerenkov spectrum [18], [19], [20] was incorporated in the model of multi-spectral diffuse light propagation. The wavelength-dependent scattering and attenuation coefficients were selected depending on the imaged tissues [44]. The reconstructed 3D images revealed that CLI signal originated from shallow locations (Fig. 6b), and that a significant portion of the signal was coming from interscapular BAT (Fig. 6a–d). This signal was particularly obvious in coronal images that clearly displayed two lobes of BAT (Fig. 6a, d). Notably, the contour was very similar to the physical triangle shape of BAT shown in Fig. 6e.

## Discussion

BAT or brown fat, widely known as ‘good fat,’ has been considered as a furnace in the body for burning excess calories. The most distinct characteristics of BAT include a large number of mitochondria, abundant UCP-1 (uncoupling protein-1) expression, and numerous small oil droplets in a single cell, as well as significant vascularization of BAT tissue [7], [10], [12], [48], [49]. These characteristics strongly indicate that BAT plays important roles in metabolism and energy expenditure under both healthy and disease status. In humans, BAT is highly abundant in embryonic and early postnatal stages, but is drastically reduced or is considered to have no physiologic relevance in adult humans [1], [2], [3]. However, the importance of BAT has recently “re-emerged” in new studies using PET imaging. PET images have shown that BAT is still present in adults in the upper chest, neck and other locations [7], [10], [12]. Recently, Cypess et al. imaged and analyzed 3,640 patients and showed that BMI (body mass index) inversely correlated to the amount of brown adipose tissue, suggesting that BAT is an important target in obesity and diabetes [7]. Other studies have also demonstrated that both BMI and body fat percentage had a significant negative correlation with BAT, whereas resting metabolic rate correlated positively with BAT [8], [17]. Moreover, it has been reported that individual differences in energy expenditure could have significant, long-term effects on body weight, and that relatively low energy expenditure predicts a weight gain [8], [17]. Recently Nagajyothi et al. suggested that BAT is one of the targets for some parasite infection [50], while Herrero et al. indicated that BAT is related to inflammation [51]. There are indications that BAT has great significance during the process of ageing [11] and that its activity is closely related to exercise [15], [16]. In addition, to improve the therapy of obesity and diabetes, increasing efforts have been put into harnessing the benefits of BAT. These efforts include the engineering and transplantation of BAT and the promotion of BAT mass by drug treatment [12], [52].

Given the importance of BAT for various diseases, we believe that new imaging techniques would be highly beneficial for its studies. Currently, the most widely used imaging method for BAT is PET imaging with ^18^F-FDG. PET and CLI imaging both have advantages and disadvantages in terms of cost, sensitivity, spatial and temporal resolution, high throughput and handiness. The advantages of CLI include its low cost imaging system, high sensitivity, potential high spatial resolution with certain radionuclides [20], capacity for high throughput, and ease of learning and usage. Its major disadvantages include tissue penetrating limitation and low sensitivity for deep targets [26], [35], [36].

For small animal imaging, the obvious benefits of CLI include a low cost of the imaging system, fast image acquisition for 2D planar imaging, and high throughput capacity. Although CLI signal from ^18^F-FDG is not very strong, the highly sensitive, cooled camera (−90°C) in optical systems could make each imaging acquisition time considerably shorter than that of PET imaging. In our case, high quality CLI images with ^18^F-FDG (7.4–11.1 MBq with i.v. injection) were obtained using a 2-minute acquisition time, while PET imaging normally required more than 5 minutes for each imaging session. However, advanced CLI techniques such as multi-spectral imaging and 3D tomography would normally need longer acquisition times (10–30 minutes). Recently, studies have indicated that the sensitivity of CLI is comparable to PET for shallow targets such as xenografted tumors, lymph nodes and the thyroid [18], [20], [34], [54]. Our data also indicated that CLI is a very sensitive method for BAT imaging both *in vivo* and *ex vivo*. CLI signal from BAT was detectable at even two hours after 10.3 MBq ^18^F-FDG injection (Fig. 1b,c) and *ex vivo* biodistribution indicated that 7.4 KBq ^18^F-FDG could produce considerable CLI signal (Fig. S1).

Biodistribution data measured by radioactivity counting and CLI were very similar. Heart and BAT were the tissues with the highest ^18^F-FDG uptake in both modalities. However, the CLI signal ratio of BAT/heart was higher than that of radioactivity counting (2.92 vs 1.76, Fig. 2a,b). This was probably due to the low CLI signal from the heart, in which a large amount of blood could absorb the emitted CLI light. CLI is a planar imaging method, which collects signals from the body surface; therefore, it is necessary to verify whether the CLI signal at the interscapular site is from BAT. Our biodistribution data indicated that the majority CLI signal at the interscapular site was from BAT, and a further BAT removal experiments validated this indication. Our correlation studies between *in vivo* CLI, *ex vivo* CLI, and radioactivity countings indicated that BAT imaging with CLI was reliable. Excellent correlation of *in vivo* CLI and *ex vivo* CLI from dissected BAT again suggested that *in vivo* CLI signal at the interscapular site was primarily from BAT. Although we did not conduct PET imaging with ^18^F-FDG to correlate with our *in vivo* CLI signal, the radioactivity counting could be considered a reasonable surrogate for PET imaging because both methods reflect the actual amount of radioactive substance in the target tissue.

In human studies, it has been reported that PET imaging signals of BAT deposits with ^18^F-FDG varied considerably under different conditions. For instance, only 3%–8% of patients’ BAT stores could be clearly visualized with 18F-FDG if no cold or drug stimulation was applied. On the contrary, after cold activation >95% of patients’ BAT could be imaged with ^18^F-FDG [7], [8]. However, we observed that this was not the case for mice, because there was an apparent CLI signal even under non-treated conditions (Fig. 1b). This is probably due to the certain level of BAT activation in mice that they maintain to keep their body warm, particularly nude mice. Spectral unmixing is a very useful technique in fluorescence imaging, which could enable the removal of autofluorescence from a subject. However, it is not often used in cases of bioluminescence imaging because bioluminescence background from non-luciferase expressing tissue is always negligible. Nonetheless, the uptake of ^18^F-FDG in mice is not highly target-specific (even though heart, BAT, and brain are the major targets) and some unspecific CLI signals are detectable. We found that this unspecific uptake problem could be solved with the spectral unmixing technique. The unmixed spectrum of unspecific ^18^F-FDG in skin and superficially in muscle was very similar to the CLI spectrum of pure ^18^F^-^ in water [18], [20]. The CLI spectrum of BAT reflected the actual surroundings of BAT, which contain abundant heme proteins, which form hemoglobin in blood and cytochrome C in mitochondria. Our 3D reconstruction was conducted with a commercial optical system using the diffusive properties of different wavelengths. The method allowed us to avoid using a complicated optical imaging system that requires acquisition of multiple-angle view images and therefore could be easily operated and accessed. The 3D images indicated that the majority of CLI signals originated from shallow locations, but not from the heart or other organs. This result was consistent with our biodistribution studies. It is widely known that 2D planar optical imaging is semi-quantitative [53], so is 2D CLI used in this report. Although 3D CLI tomography imaging could provide better quantification, and great efforts have been put into different reconstruction methods [31], [45], [54], commercially available software packages are still not available yet. Therefore the accessibility of 3D CLI is the bottleneck for wide application of this advanced technology. Hopefully, this limitation could probably be overcome in the near future.

For the very recent development of this technology, it is very exciting that the first human CLI imaging has been successfully demonstrated [55]. Conceivably, CLI imaging of BAT in the neck area in humans is possible due to its shallow location.

In summary, we demonstrated that CLI with ^18^F-FDG could be used as a reliable method to image BAT and to monitor its activation. This method is particularly useful for small animal models where BAT location is ideal for optical registration. Compared to ^18^F-FDG PET imaging, CLI is much cheaper, faster, and can be used for *in vivo* high throughput screening as well. In addition, our study also showed that 3D tomography of CLI is feasible and that 3D volume quantification is possible for future studies. For small animal imaging, we believe that CLI imaging of BAT could be a useful complementary tool to PET imaging in small animals for researchers investigating BAT.

## Experiments

Female nu/nu mice 4–8 weeks of age were purchased from Massachusetts General Hospital Radiation Oncology breeding facilities. All experimental procedures were approved by the Subcommittee on Research Animal Care at Massachusetts General Hospital. CLI imaging was performed using an IVIS®Spectrum animal imaging system (Perkin Elmer/Caliper LifeSciences, Hopkinton, MA). Image analysis was conducted using LivingImage® 4.2 software. The ^18^F-FDG images were not decay corrected for all quantification because our comparisons between different animal groups were made at the same time-points, and acquisition parameters were the same. ^18^F-FDG was purchased from IBA Molecular.

### 1. Feasibility Study

Mice (n = 3) were anesthetized with isoflurane balanced with oxygen for 5 minutes and injected with 10.3 MBq of ^18^F-FDG intravenously. Injected mice were then subjected to luminescence imaging at 30, 60, and 120 minutes after ^18^F-FDG injection with the following parameters: open filter, f = 1, bin = 8, FOV = D, and exposure time = 120 s.

### 2. Validation of BAT Location by CLI and Biodistribution Studies

Mice (n = 3) were anesthetized with isoflurane balanced with oxygen for 5 minutes and injected with 10.3 MBq of ^18^F-FDG intravenously. Mice were sacrificed at 60 minutes after injection. The skin from the interscapular area was removed and mice underwent imaging before and after BAT removal, using the same parameters as listed above in the feasibility study section. After imaging, organs were dissected, collected and weighed, and subjected to optical imaging (open filter, f = 1, bin = 8, FOV = D, and exposure time = 120 s) and to radioactivity counting with dosimeters. The distribution of signals was normalized to the weight of the tested organs.

### 3. Reliability Studies with Ketamine Anesthesia and NE Treatment

Nude mice (n = 8) were divided into two groups (n = 4 each). One group was injected with norepinephrine (NE) (50 uL, 10 mM) intraperitoneally. The second group served as a non-injected control. After 30 minutes, both groups were injected intraperitoneally with ketamine/xylazine and kept under anesthesia for the following 60–70 minutes at room temperature. Once mice were anesthetized, ^18^F-FDG (8.1 MBq) was intravenously injected into each mouse. Mice were imaged at 60 minutes after ^18^F-FDG injections, using the same parameters as for the feasibility study. For the correlation study, mice were sacrificed immediately after imaging, BAT was excised, and CLI imaging and a dosimeter counting were performed.

### 4. Monitoring Activation with NE and Cold Exposure Under Isoflurane Anesthesia

A similar procedure for NE activation and cold exposure was followed as above, except isoflurane was used and anesthesia time lasted for 5 minutes before each image acquisition (n = 4). For long isoflurane anesthesia (60 minutes), mice were kept in an isoflurane induction chamber after ^18^F-FDG injection. For cold exposure study, mice were placed in a cold room (4°C) for 4 hours before ^18^F-FDG injection and returned to the cold room once recovered from anesthesia until the images were acquired. The body temperatures of the mice in the cold room were measured with a rectal thermometer and we found that their temperatures were about 30°C.

### 5. Spectral Unmixing and Multispectral Cerenkov Luminescence Tomography Studies

Mice were anesthetized for 5 minutes with isoflurane followed by intravenous injection of 11.1MBq ^18^F-FDG. Multispectral images were acquired with the following parameters: f = 1, bin = 16, acquisition time = 300s per filter, emission filters = 580, 600, 620, 640, 660 and 680 nm (raw images are shown in Fig. S2). Spectral unmixing was conducted with Living Imaging 4.31 software from Caliper, a Perkin Elmer Company, and two components and automatic unmixing were used. A 3D reconstruction was conducted according to the method reported by Kuo et al. [31], [44] and using in-house software from Caliper, a Perkin Elmer Company. The Cerenkov emission spectrum was incorporated into the model of diffuse light propagation. Tikhonov regularization was applied in the non-negative least squares optimization of the residuals, and the surface tomography generated from structure light imaging was used for 3D image co-registration.

## Supporting Information

Figure S1 **Representative CLI images of dissected tissues (n = 3).** 1) liver, 2) heart, 3) lung, 4) brain, 5) muscle, 6) kidney, 7) spleen, 8) intestine, 9) bladder, 10) skin, 11) blood, 12) stomach, 13) white fat from belly, 14) BAT.
(TIF)

Figure S2 **Quantitative analysis of signals of the NE-treated group and the control group.** Radioactivity reading (a), *in vivo* CLI signals (b), and *ex vivo* CLI signals (c) (n = 4 for each group).
(TIF)

Figure S3 **Raw images for spectral unmixing and multispectral Cerenkov luminescence tomography imaging.**
(TIF)

## References

[1] Cannon B, Nedergaard J (2004) Brown adipose tissue: function and physiological significance. Physiol Rev 84: 277–359. 14715917 10.1152/physrev.00015.2003

[2] Richard D, Picard F (2011) Brown fat biology and thermogenesis. Frontiers Biosci 16: 1233–1260. 10.2741/3786 21196229

[3] Ouellet V, Labbe SM, Blondin DP, Phoenix S, Guerin B, et al (2012) Brown adipose tissue oxidative metabolism contributes to energy expenditure during acute cold exposure in humans. J. Clin Invest 122: 545–552. 22269323 10.1172/JCI60433 PMC3266793

[4] Lowell BB, Flier JS (1997) Brown adipose tissue, beta 3-adrenergic receptors, and obesity. Ann Rev Med 48: 307–316. 9046964 10.1146/annurev.med.48.1.307

[5] Stephens M, Ludgate M, Rees DA (2011) Brown fat and obesity: the next big thing? Clin Endocrinol 74: 661–670. 10.1111/j.1365-2265.2011.04018.x 21521287

[6] Koppen A, Kalkhoven E (2010) Brown vs white adipocytes: the PPARgamma coregulator story. FEBS Lett 584: 3250–3259. 20600006 10.1016/j.febslet.2010.06.035

[7] Cypess AM, Lehman S, Williams G, Tal I, Rodman D, et al (2009) Identification and importance of brown adipose tissue in adult humans. New Engl J Med 360: 1509–1517. 19357406 10.1056/NEJMoa0810780 PMC2859951

[8] van Marken Lichtenbelt WD, Vanhommerig JW, Smulders NM, Drossaerts JM, Kemerink GJ, et al (2009) Cold-activated brown adipose tissue in healthy men. New Engl J Med 360: 1500–1508. 19357405 10.1056/NEJMoa0808718

[9] Zingaretti MC, Crosta F, Vitali A, Guerrieri M, Frontini A, et al (2009) The presence of UCP1 demonstrates that metabolically active adipose tissue in the neck of adult humans truly represents brown adipose tissue. FASEB J 23: 3113–3120. 19417078 10.1096/fj.09-133546

[10] Nedergaard J, Bengtsson T, Cannon B (2007) Unexpected evidence for active brown adipose tissue in adult humans. Amer J Physiol 293: E444–452. 10.1152/ajpendo.00691.2006 17473055

[11] Mattson MP (2010) Perspective: Does brown fat protect against diseases of aging? Ageing Res Rev 9: 69–76. 19969105 10.1016/j.arr.2009.11.004 PMC2818667

[12] Tran TT, Kahn CR (2010) Transplantation of adipose tissue and stem cells: role in metabolism and disease. Nat Rev Endocrinol 6: 195–213. 20195269 10.1038/nrendo.2010.20 PMC4362513

[13] Basu S (2008) Functional imaging of brown adipose tissue with PET: can this provide new insights into the pathophysiology of obesity and thereby direct antiobesity strategies? Nucl Med Commun 29: 931–933. 18836369 10.1097/MNM.0b013e328310af46

[14] Chen YI, Cypess AM, Sass CA, Brownell AL, Jokivarsi KT, et al (2012) Anatomical and Functional Assessment of Brown Adipose Tissue by Magnetic Resonance Imaging. Obesity 20: 1519–1526. 22343821 10.1038/oby.2012.22 PMC4383098

[15] Bostrom P, Wu J, Jedrychowski MP, Korde A, Ye L, et al (2012) A PGC1-alpha-dependent myokine that drives brown-fat-like development of white fat and thermogenesis. Nature 481: 463–468. 22237023 10.1038/nature10777 PMC3522098

[16] Xu X, Ying Z, Cai M, Xu Z, Li Y, et al (2011) Exercise ameliorates high-fat diet-induced metabolic and vascular dysfunction, and increases adipocyte progenitor cell population in brown adipose tissue. Am J Physiol Regul 300: R1115–1125. 10.1152/ajpregu.00806.2010 PMC3094041 21368268

[17] Yoneshiro T, Aita S, Matsushita M, Okamatsu-Ogura Y, Kameya T, et al (2011) Age-related decrease in cold-activated brown adipose tissue and accumulation of body fat in healthy humans. Obesity 19: 1755–1760. 21566561 10.1038/oby.2011.125

[18] Robertson R, Germanos MS, Li C, Mitchell GS, Cherry SR, et al (2009) Optical imaging of Cerenkov light generation from positron-emitting radiotracers. Phys Med Biol 54: N355–365. 19636082 10.1088/0031-9155/54/16/N01 PMC2765256

[19] Spinelli AE, D’Ambrosio D, Calderan L, Marengo M, Sbarbati A, et al (2010) Cerenkov radiation allows in vivo optical imaging of positron emitting radiotracers. Phys Med Biol 55: 483–495. 20023328 10.1088/0031-9155/55/2/010

[20] Liu H, Ren G, Miao Z, Zhang X, Tang X, et al (2010) Molecular optical imaging with radioactive probes. PloS one 5: e9470. 20208993 10.1371/journal.pone.0009470 PMC2830426

[21] Ran C, Zhang Z, Hooker J, Moore A (2012) In vivo photoactivation without “light”: use of Cherenkov radiation to overcome the penetration limit of light. Mol Imag Biol 14: 156–162. 10.1007/s11307-011-0489-z 21538154

[22] Lucignani G (2011) Cerenkov radioactive optical imaging: a promising new strategy. Eur J Nucl Med Mol Imag 38: 592–595. 10.1007/s00259-010-1708-6 21174087

[23] Dothager RS, Goiffon RJ, Jackson E, Harpstrite S, Piwnica-Worms D (2010) Cerenkov radiation energy transfer (CRET) imaging: a novel method for optical imaging of PET isotopes in biological systems. PloS one 5: e13300. 20949021 10.1371/journal.pone.0013300 PMC2952622

[24] Ruggiero A, Holland JP, Lewis JS, Grimm J (2010) Cerenkov luminescence imaging of medical isotopes. J Nucl Med 51: 1123–1130. 20554722 10.2967/jnumed.110.076521 PMC3068779

[25] Lewis MA, Kodibagkar VD, Oz OK, Mason RP (2010) On the potential for molecular imaging with Cerenkov luminescence. Optics Letters 35: 3889–3891. 21124555 10.1364/OL.35.003889 PMC3023798

[26] Mitchell GS, Gill RK, Boucher DL, Li C, Cherry SR (2011) In vivo Cerenkov luminescence imaging: a new tool for molecular imaging. Phil Trans Series A, Math, Physi Eng Sci 369: 4605–4619. 10.1098/rsta.2011.0271 PMC3263789 22006909

[27] Spinelli AE, Boschi F (2012) Optimizing in vivo small animal Cerenkov luminescence imaging. J Biomed Optics 17: 040506. 10.1117/1.JBO.17.4.040506 22559672

[28] Cherenkov PA (1934) Visible emission of clean liquids by action of γ radiation Doklady Akademii Nauk SSSR. 2: 451.

[29] Liu H, Zhang X, Xing B, Han P, Gambhir SS, et al (2010) Radiation-luminescence-excited quantum dots for in vivo multiplexed optical imaging. Small 6: 1087–1091. 20473988 10.1002/smll.200902408

[30] Holland JP, Normand G, Ruggiero A, Lewis JS, Grimm J (2012) Intraoperative imaging of positron emission tomographic radiotracers using cerenkov luminescence emissions. Mol Imag 11: 1–10. PMC3083828 21496448

[31] Spinelli AE, Kuo C, Rice BW, Calandrino R, Marzola P, et al (2011) Multispectral Cerenkov luminescence tomography for small animal optical imaging. Optics Express 19: 12605–12618. 21716501 10.1364/OE.19.012605

[32] Xu Y, Chang E, Liu H, Jiang H, Gambhir SS, et al (2012) Proof-of-concept study of monitoring cancer drug therapy with cerenkov luminescence imaging. J Nucl Med 53: 312–317. 22241909 10.2967/jnumed.111.094623 PMC4143153

[33] Liu H, Carpenter CM, Jiang H, Pratx G, Sun C, et al (2012) Intraoperative imaging of tumors using cerenkov luminescence endoscopy: a feasibility experimental study. J Nucl Med 53: 1579–1584. 22904353 10.2967/jnumed.111.098541 PMC4887274

[34] Thorek DL, Abou DS, Beattie BJ, Bartlett RM, Huang R, et al (2012) Positron lymphography: multimodal, high-resolution, dynamic mapping and resection of lymph nodes after intradermal injection of 18F-FDG. J Nucl Med 53: 1438–1445. 22872741 10.2967/jnumed.112.104349 PMC3537831

[35] Xu Y, Liu H, Cheng Z (2011) Harnessing the power of radionuclides for optical imaging: Cerenkov luminescence imaging. J Nucl Med 52: 2009–2018. 22080446 10.2967/jnumed.111.092965

[36] Spinelli AE, Marengo M, Calandrino R, Sbarbati A, Boschi F (2012) Optical imaging of radioisotopes: a novel multimodal approach to molecular imaging. Quart J Nucl Med Mol Imag 56: 280–290. 22695338

[37] Boschi F, Meo SL, Rossi PL, Calandrino R, Sbarbati A, et al (2011) Optical imaging of alpha emitters: simulations, phantom, and in vivo results. J Biomed Optics 16: 126011. 10.1117/1.3663441 22191928

[38] Ackerman NL, Graves EE (2012) The potential for Cerenkov luminescence imaging of alpha-emitting radionuclides. Phys Med Biol 57: 771–783. 22252144 10.1088/0031-9155/57/3/771 PMC5558792

[39] Tatsumi M, Engles JM, Ishimori T, Nicely O, Cohade C, et al (2004) Intense (18)F-FDG uptake in brown fat can be reduced pharmacologically. J Nucl Med 45: 1189–1193. 15235065

[40] Wu C, Cheng W, Xing H, Dang Y, Li F, et al (2011) Brown adipose tissue can be activated or inhibited within an hour before 18F-FDG injection: a preliminary study with microPET. J Biomed Biotech 2011: 159834. 10.1155/2011/159834 PMC3085214 21541240

[41] Fueger BJ, Czernin J, Hildebrandt I, Tran C, Halpern BS, et al (2006) Impact of animal handling on the results of 18F-FDG PET studies in mice. J Nucl Med 47: 999–1006. 16741310

[42] Ocloo A, Shabalina IG, Nedergaard J, Brand MD (2007) Cold-induced alterations of phospholipid fatty acyl composition in brown adipose tissue mitochondria are independent of uncoupling protein-1. Am J Physiol Regul 293: R1086–1093. 10.1152/ajpregu.00128.2007 17609311

[43] Ohlson KB, Shabalina IG, Lennstrom K, Backlund EC, Mohell N, et al (2004) Inhibitory effects of halothane on the thermogenic pathway in brown adipocytes: localization to adenylyl cyclase and mitochondrial fatty acid oxidation. Biochem Pharmacol 68: 463–477. 15242813 10.1016/j.bcp.2004.03.028

[44] Kuo C, Coquoz O, Troy TL, Xu H, Rice BW (2007) Three-dimensional reconstruction of in vivo bioluminescent sources based on multispectral imaging. J Biomed Optics 12: 024007. 10.1117/1.2717898 17477722

[45] Li C, Mitchell GS, Cherry SR (2010) Cerenkov luminescence tomography for small-animal imaging. Optics letters 35: 1109–1111. 20364233 10.1364/OL.35.001109 PMC2852688

[46] Zhong J, Qin C, Yang X, Chen Z, Tian J (2011) Fast-Specific Tomography Imaging via Cerenkov Emission. Mol Imag Biol 14: 286–292. 10.1007/s11307-011-0510-6 21786071

[47] Zhong J, Tian J, Yang X, Qin C (2011) L1-regularized Cerenkov luminescence tomography with a SP3 method and CT fusion. Conference proceedings : Ann Inter Confer of IEEE Eng Med Biol Soc 2011: 6158–6161. 10.1109/IEMBS.2011.6091521 22255745

[48] Tseng YH, Kokkotou E, Schulz TJ, Huang TL, Winnay JN, et al (2008) New role of bone morphogenetic protein 7 in brown adipogenesis and energy expenditure. Nature 454: 1000–1004. 18719589 10.1038/nature07221 PMC2745972

[49] Zhang H, Schulz TJ, Espinoza DO, Huang TL, Emanuelli B, et al (2010) Cross talk between insulin and bone morphogenetic protein signaling systems in brown adipogenesis. Mol Cellular Biol 30: 4224–4233. 20584981 10.1128/MCB.00363-10 PMC2937545

[50] Nagajyothi F, Desruisseaux MS, Machado FS, Upadhya R, Zhao D, et al (2012) Response of adipose tissue to early infection with Trypanosoma cruzi (Brazil strain). J Infect Dis 205: 830–840. 22293433 10.1093/infdis/jir840 PMC3274374

[51] Herrero L, Shapiro H, Nayer A, Lee J, Shoelson SE (2010) Inflammation and adipose tissue macrophages in lipodystrophic mice. Proc Natl Acad Sci USA107: 240–245. 10.1073/pnas.0905310107 PMC2806777 20007767

[52] Gunawardana SC, Piston DW (2012) Reversal of type 1 diabetes in mice by brown adipose tissue transplant. Diabetes 61: 674–682. 22315305 10.2337/db11-0510 PMC3282804

[53] Massoud TF, Gambhir SS (2003) Molecular imaging in living subjects: seeing fundamental biological processes in a new light. Genes & Develop 17: 545–580. 12629038 10.1101/gad.1047403

[54] Hu Z, Ma X, Qu X, Yang W, Liang J, et al (2012) Three-dimensional noninvasive monitoring iodine-131 uptake in the thyroid using a modified Cerenkov luminescence tomography approach. PloS one 7: e37623. 22629431 10.1371/journal.pone.0037623 PMC3358266

[55] Spinelli AE, Ferdeghini M, Cavedon C, Zivelonghi E, Calandrino R, et al (2013) First human Cerenkography. J Biomed Optics 18: 20502. 10.1117/1.JBO.18.2.020502 23334715

