# Supplementary figures and images for: In Vivo Optical Imaging of Interscapular Brown Adipose Tissue with 18F-FDG via Cerenkov Luminescence Imaging
Source: PLoS One. 2013 Apr 24;8(4):e62007. doi: 10.1371/journal.pone.0062007 (PMC3634850; doi:10.1371/journal.pone.0062007)

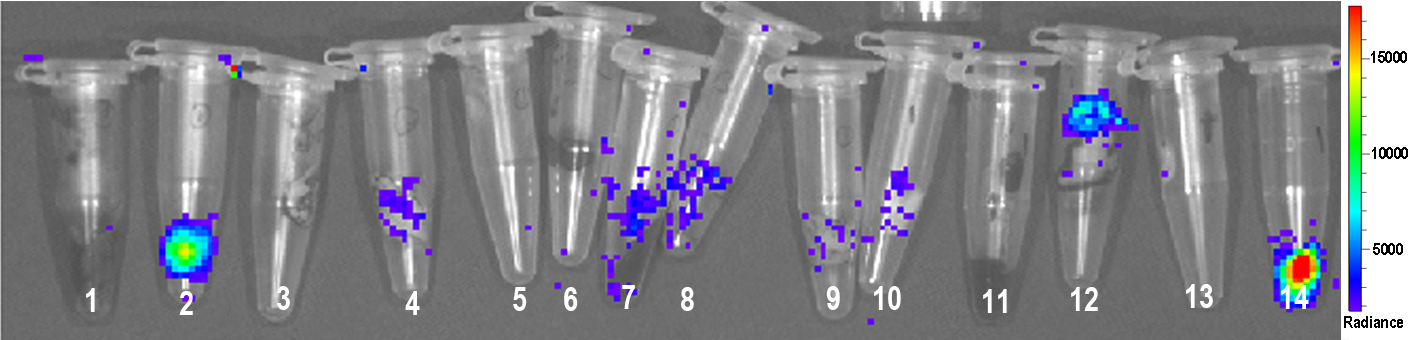

Supplement: Figure S1 — Representative CLI images of dissected tissues (n = 3). 1) liver, 2) heart, 3) lung, 4) brain, 5) muscle, 6) kidney, 7) spleen, 8) intestine, 9) bladder, 10) skin, 11) blood, 12) stomach, 13) white fat from belly, 14) BAT. (TIF) [file pone.0062007.s001.tif]

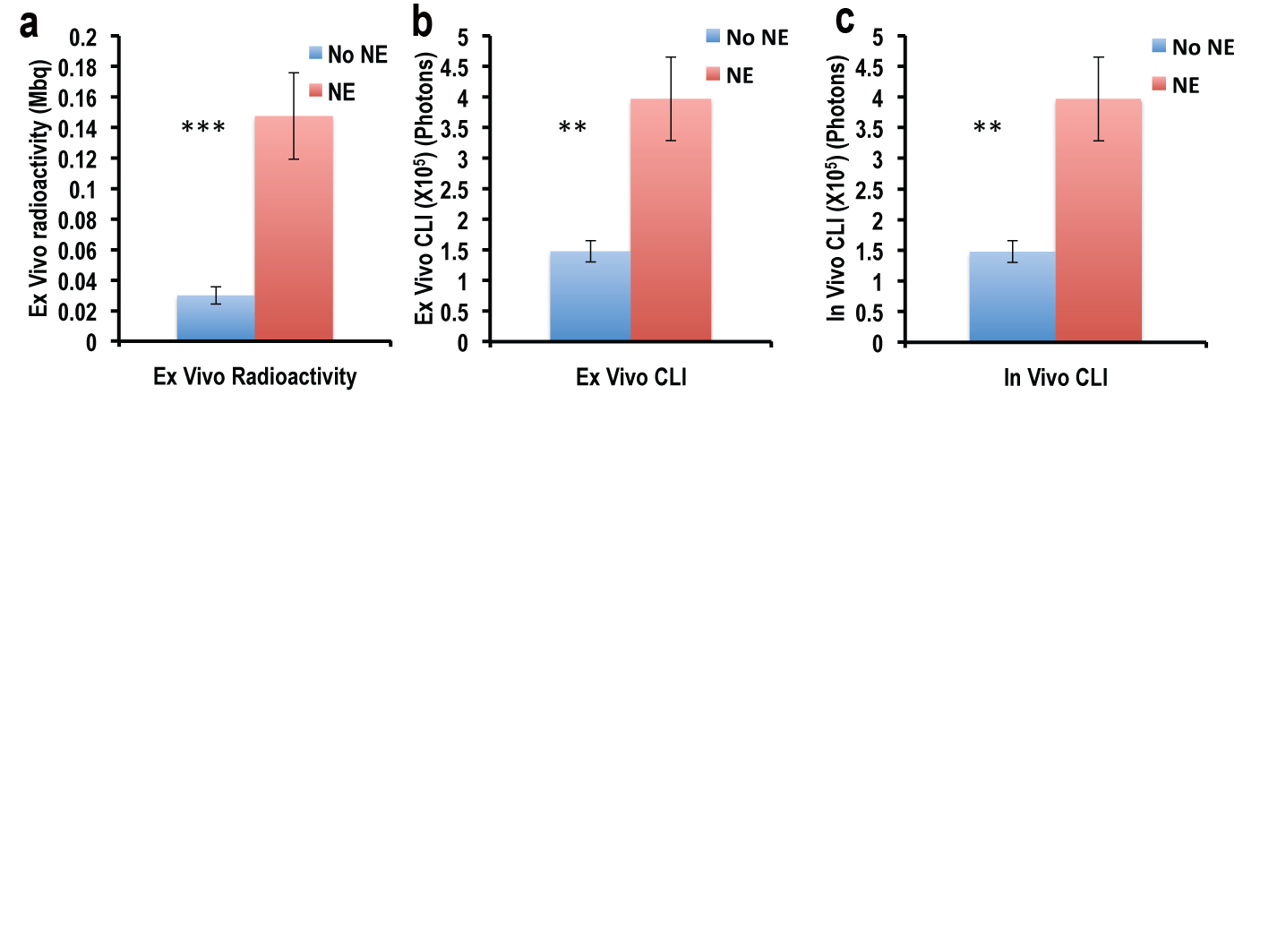

Supplement: Figure S2 — Quantitative analysis of signals of the NE-treated group and the control group. Radioactivity reading (a), in vivo CLI signals (b), and ex vivo CLI signals (c) (n = 4 for each group). (TIF) [file pone.0062007.s002.tif]

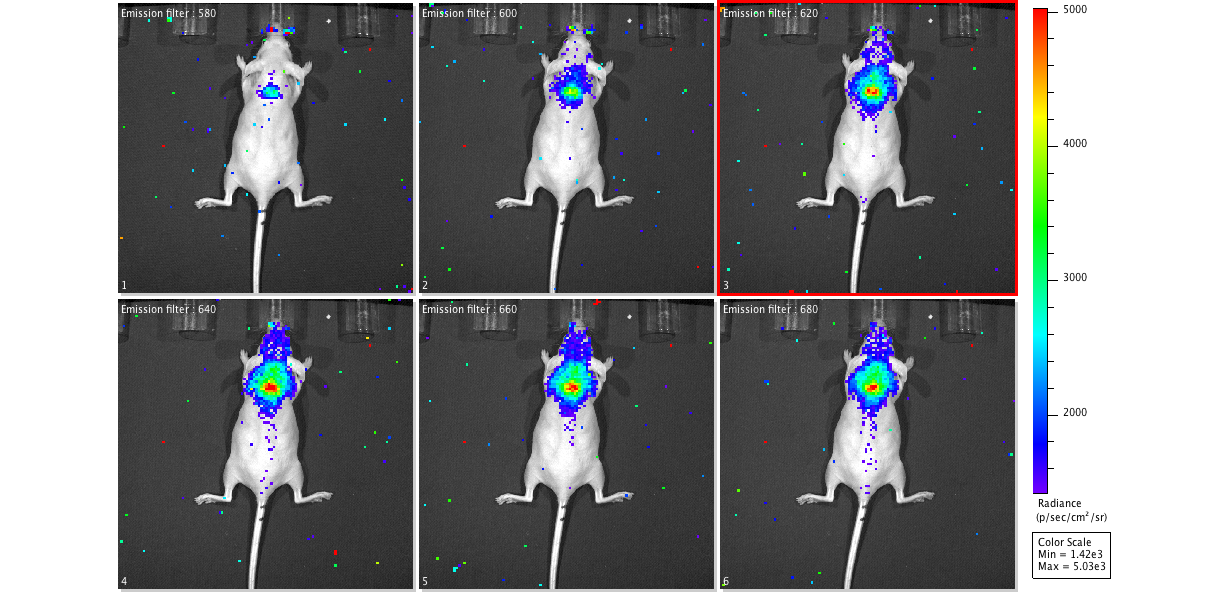

Supplement: Figure S3 — Raw images for spectral unmixing and multispectral Cerenkov luminescence tomography imaging. (TIF) [file pone.0062007.s003.tif]
